# Large Bowel Internal Hernia Secondary to Reimplanted Ureter

**DOI:** 10.1155/2024/2061453

**Published:** 2024-04-30

**Authors:** Amy A. Howk, Anna Hayden, John E. Griepentrog

**Affiliations:** ^1^Department of General Surgery, University of Tennessee Medical Center, Knoxville, TN, USA; ^2^University of South Carolina School of Medicine, Greenville, SC, USA

## Abstract

Internal hernias are difficult to diagnose clinically, and normal cross-sectional imaging has been documented in many patients. Transmesenteric hernias from congenital defects or prior abdominal surgery are most common. A 46-year-old previously healthy female presented to the emergency department with acute onset nausea and vomiting eight years after a right ureteral transection during a laparoscopic hysterectomy, with a delayed ureterolysis and reimplantation into the bladder. Rectal contrast CT scan demonstrated a partial large bowel obstruction that was unclear if it was due to an underlying mass, stricture, or internal herniation. The patient was taken for exploratory laparotomy which demonstrated redundant transverse colon herniated under the mobile right ureter; an extended right hemicolectomy was performed. This report describes alteration of retroperitoneal anatomy creating a potential space for colonic herniation and emphasizes that clinical suspicion must remain high for patients presenting with obstructive or partially obstructive symptoms.

## 1. Introduction

Internal hernias are notoriously difficult to diagnose clinically. Transmesenteric hernias are the most common type, resulting from congenital mesenteric defects or prior abdominal surgery. CT scan allows for definitive diagnosis in most cases. However, normal cross-sectional imaging has been documented in up to twenty percent of patients with surgically confirmed internal hernias [1]. Internal hernias can be caused by defects in the mesentery, adhesive peritoneal bands, and omental adhesions and can result in volvulus and strangulation [2, 3]. This case represents a non-transmesenteric hernia involving the right ureter secondary to altered retroperitoneal anatomy. This case report is unique due to the non-transmesenteric location and the delayed time to presentation after the ureter reimplantation (approximately eight years). This case report was written using the SCARE 2023 guidelines [4]. Written consent was obtained from the patient to submit this case report.

## 2. Case Presentation

The patient is a 46-year-old female who presented to the emergency department with nonlocalized abdominal pain and constipation intermittently for the past two weeks. Her pain worsened on the day of presentation with new onset nausea and vomiting. Her family history was significant for colon polyps, with her last colonoscopy eight years prior without abnormality. Her surgical history included a right ureteral transection during a laparoscopic hysterectomy for which she had undergone delayed repair with ureterolysis and reimplantation into the bladder dome eight years prior.

On examination, the patient was nontoxic appearing with stable vital signs. Her abdominal exam was soft, somewhat distended, with mild tenderness and no peritoneal signs. CT of the abdomen and pelvis with intravenous contrast demonstrated dense impacted mid colonic stool at the distal transverse colon with mild inflammation and abrupt tapering, as well as severe right hydronephrosis (Figure S1, Figure S2). Her initial creatinine was elevated at 1.37. Rectal contrast CT scan demonstrated a partial large bowel obstruction. It was unclear if her colonic obstruction was due to an underlying mass, stricture, or internal herniation. Colonoscopy revealed no mass but abnormal luminal narrowing concerning for volvulus. Endoscopic detorsion was attempted and unsuccessful.

The patient was taken for exploratory laparotomy, and the redundant transverse colon was found to be herniated behind the mobile right ureter (Figure S3). The colon was reduced with gentle traction, and the bowel appeared viable. The mid transverse colon was strictured without evidence of a mass. Due to the stricturing and redundancy of the transverse colon, an extended right hemicolectomy with stapled anastomosis was performed. The ureter was evaluated and noted to be severely dilated and tortuous (Figure S4). A distal ureterectomy with removal of bladder cuff and ureteroneocystostomy were then performed at that time.

She was continued on maintenance fluids and trialed a regular diet following her procedures. Her creatinine normalized to 0.7 postoperatively. Her postoperative course was significant for an ileus and an intra-abdominal abscess requiring percutaneous drainage (Figure S5). The abscess was discovered on a CT scan obtained on postoperative day five to investigate her ileus and a leukocytosis. Following drain placement, she was able to tolerate a regular diet. She was discharged home on postoperative day eight. She has since followed up in the general surgery clinic and urology clinic. At four-week follow-up, CT of the abdomen and pelvis was obtained that demonstrated no residual intra-abdominal abscess and no evidence of hydronephrosis. The drain was removed. She has normal bowel function and no issues with her ureter or renal function at six-month follow-up.

## 3. Discussion

Internal hernias account for up to 5% of all cases of intestinal obstruction [2]. Internal hernias are most commonly congenital (paraduodenal, pericecal, foramen of Winslow, transmesenteric, transomental, and sigmoid mesocolon); however, acquired internal hernias can occur following alterations in the mesentery via surgical procedure or trauma, as well as a result of inflammation [3]. Acquired hernias typically occur through mesenteric defects after bowel resection or Roux-en-Y anastomosis [3, 5, 6]. The incidence of internal hernias has increased as more Roux-en-Y gastric bypass procedures are being performed [2, 6]. As alteration of normal anatomy via surgical procedures becomes more frequent, clinical suspicion must remain high for patients presenting with obstructive or partially obstructive symptoms. This patient had altered anatomy that created an uncommon potential space for visceral organ herniation.

Ureteral injury is a common complication of pelvic operations, with incidence ranging from 1 to 10% [7]. The majority of these injuries occur at the distal ureter below the level of the pelvic brim [7]. The more distal the injury, the higher likelihood of requiring a ureteroneocystostomy. This requires mobilization of the ureter and the bladder to allow a tension-free anastomosis. In this patient's previous repair, her ureter was medialized and reimplanted into the dome of the bladder. This mobilization of the ureter from the retroperitoneum created a space that her redundant transverse colon could herniate through.

Though internal hernias appear to be well studied, most of the research focuses on small bowel obstructions with few case reports describing herniation of the colon [1, 3, 5]. Most internal hernias studied are secondary to mesenteric defects from congenital abnormalities or surgical procedures [1, 3, 5]. Reports of internal hernias without a mesenteric defect are attributed to adhesive peritoneal bands or omental adhesions [5]. This patient presented with a novel non-transmesenteric internal hernia caused by her previously surgically altered retroperitoneal anatomy.

Obstructed internal hernias can be difficult to diagnose, as symptoms can be vague and present with one or more clinical features that can vary between patients [3]. While one patient may present with vomiting, constipation, abdominal pain, and distention, the next patient may have symptoms that present more of a vague picture that could clinically worsen at any point. Additionally, the literature shows that up to twenty percent of internal hernias appear normal on cross-sectional imaging. As we did not find any literature specifically describing a case non-transmesenteric herniation of the colon through altered retroperitoneal anatomy, the strength of this case report is its novelty. The surgical approach taken was necessary, as an extended right hemicolectomy was required secondary to redundancy of the colon as well as patchy ischemia. Therefore, the main conclusion from this case report is that clinicians must maintain a high index of suspicion for patients presenting with such symptoms.

## Data Availability

The patient clinical data used to support the findings of this study are included within the article.

## Supplementary Materials

Supplementary 1 Figure S1: preoperative CT demonstrating large bowel obstruction at the transverse colon. (Arrow indicates transition point.)

Supplementary 2 Figure S2: preoperative CT with right-sided hydronephrosis.

Supplementary 3 Figure S3: transverse colon herniated posterior to reimplanted right ureter causing bowel obstruction. (Arrow indicates area of transition point behind ureter.)

Supplementary 4 Figure S4: tortuous right ureter after reduction of transverse colon.

Supplementary 5 Figure S5: postoperative CT with intra-abdominal abscess. (Arrow indicates fluid collection.)
