# Supplementary figures and images for: Large Bowel Internal Hernia Secondary to Reimplanted Ureter
Source: Case Rep Surg. 2024 Apr 30;2024:2061453. doi: 10.1155/2024/2061453 (PMC11074864; doi:10.1155/2024/2061453)

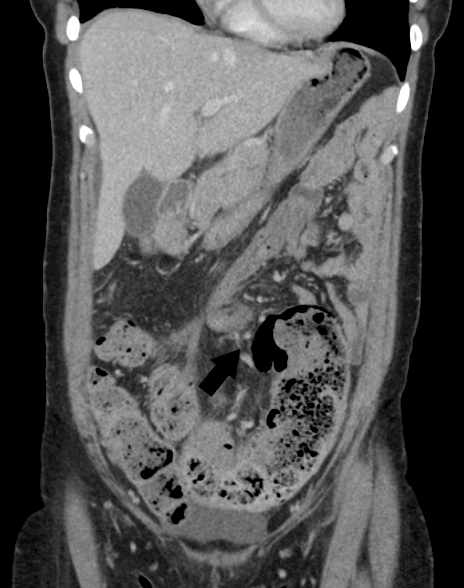

Supplement: Supplementary 1 — Figure S1: preoperative CT demonstrating large bowel obstruction at the transverse colon. (Arrow indicates transition point.) [file 2061453.f1.png]

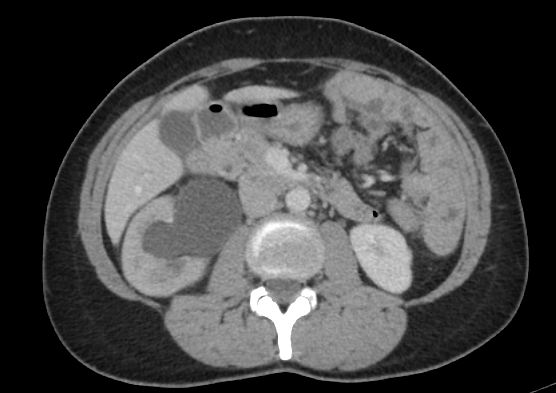

Supplement: Supplementary 2 — Figure S2: preoperative CT with right-sided hydronephrosis. [file 2061453.f2.PNG]

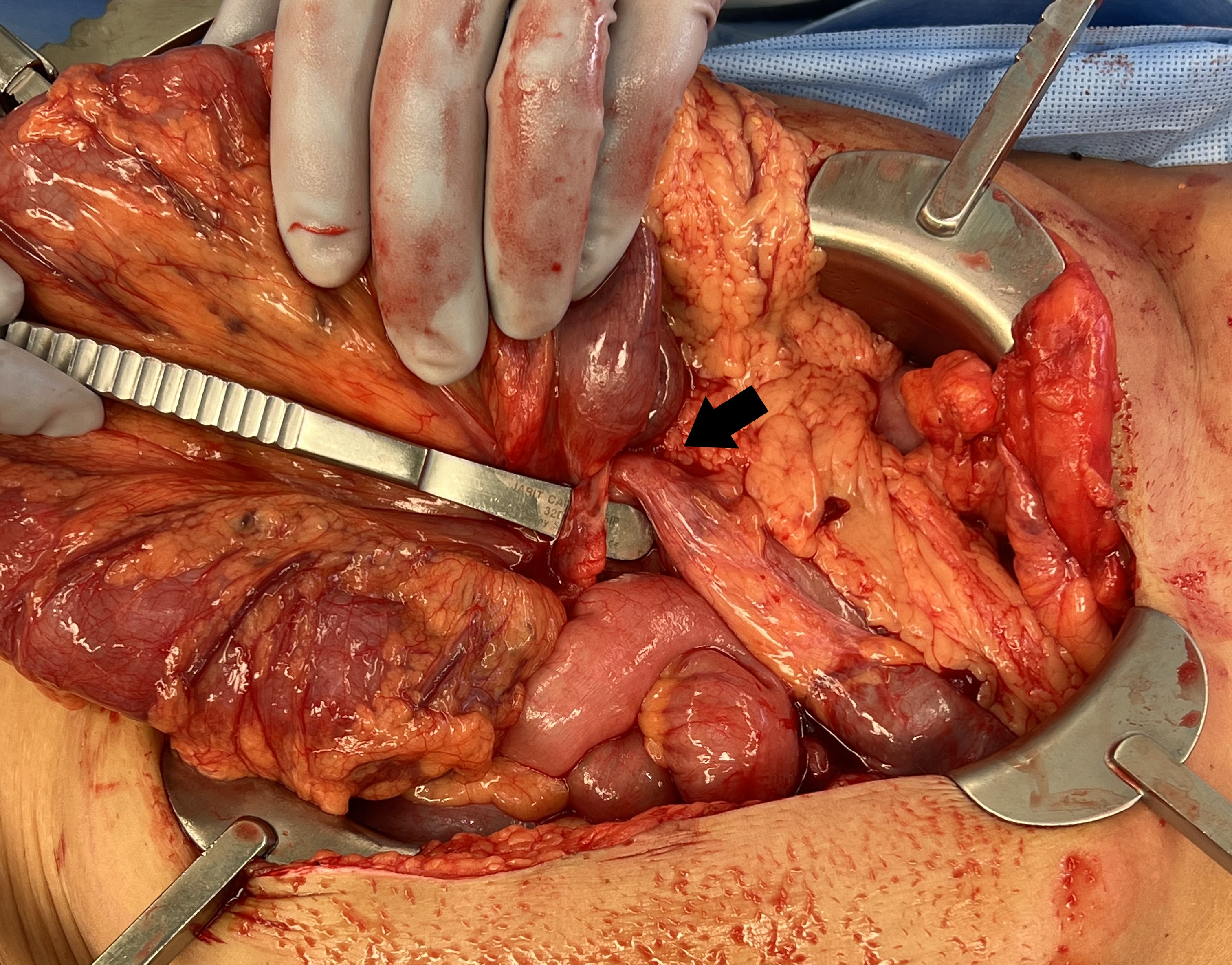

Supplement: Supplementary 3 — Figure S3: transverse colon herniated posterior to reimplanted right ureter causing bowel obstruction. (Arrow indicates area of transition point behind ureter.) [file 2061453.f3.jpg]

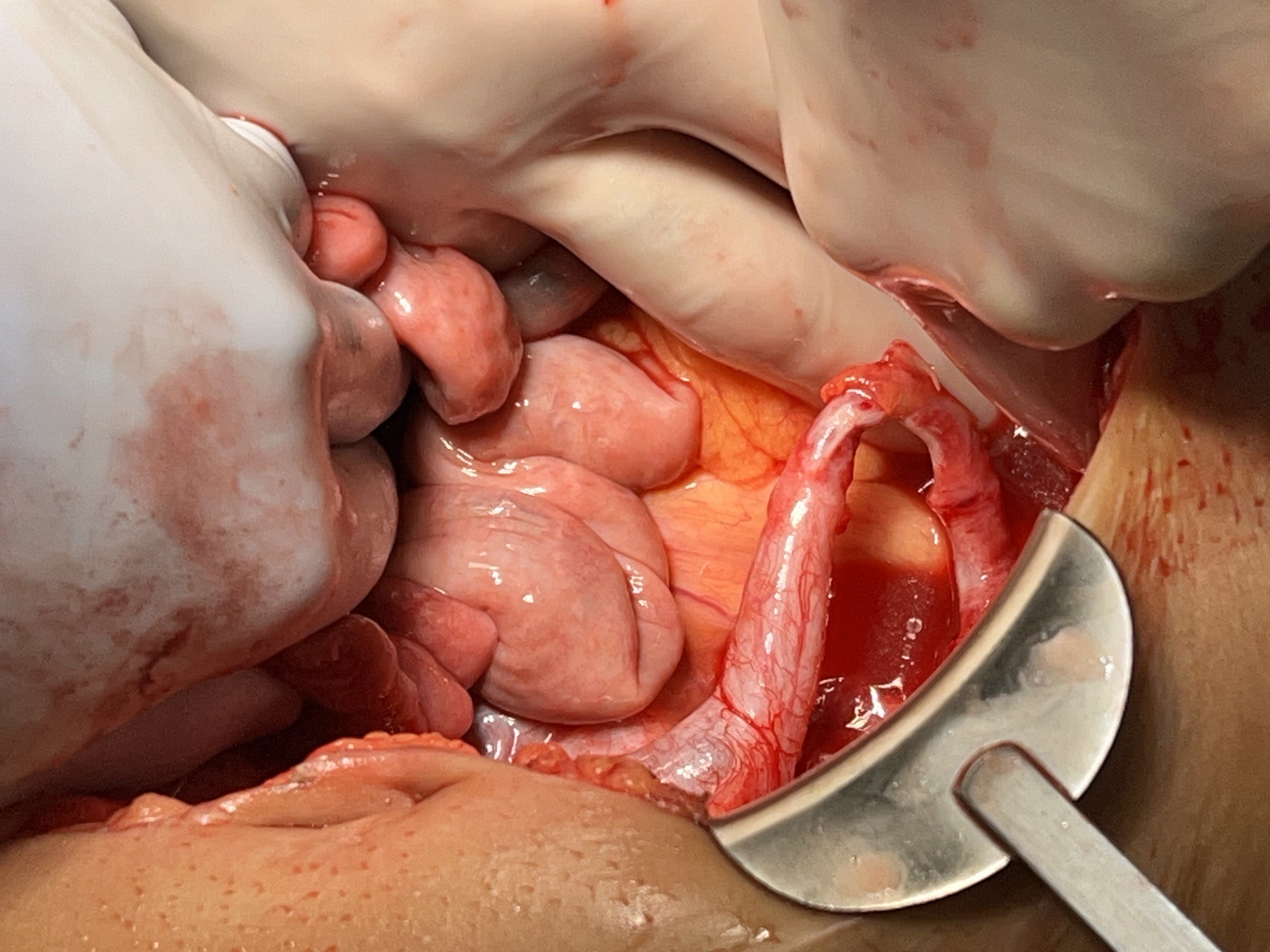

Supplement: Supplementary 4 — Figure S4: tortuous right ureter after reduction of transverse colon. [file 2061453.f4.jpeg]

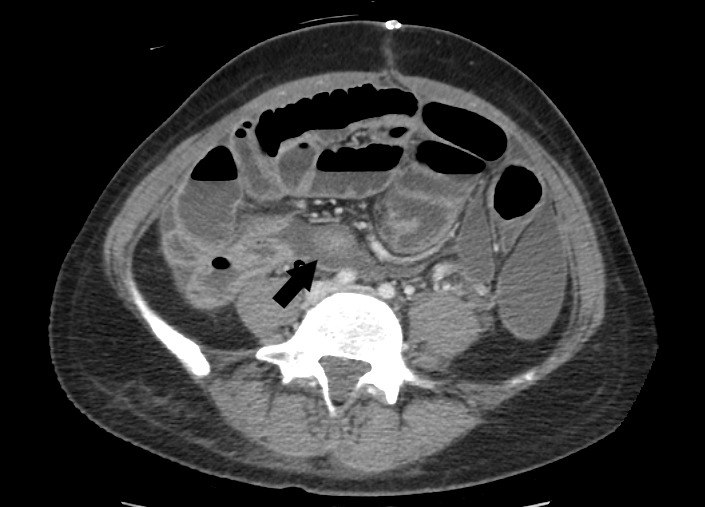

Supplement: Supplementary 5 — Figure S5: postoperative CT with intra-abdominal abscess. (Arrow indicates fluid collection.) [file 2061453.f5.png]
